# Stability of the splay state in networks of pulse-coupled neurons

**DOI:** 10.1186/2190-8567-2-12

**Published:** 2012-11-22

**Authors:** Simona Olmi, Antonio Politi, Alessandro Torcini

**Affiliations:** 1 Istituto dei Sistemi Complessi, CNR - Consiglio Nazionale delle Ricerche, via Madonna del Piano 10, I-50019, Sesto Fiorentino, Italy; 2 Centro Interdipartimentale per lo Studio delle Dinamiche Complesse, via Sansone, 1 - I-50019, Sesto Fiorentino, Italy; 3 Institute for Complex Systems and Mathematical Biology, King’s College, University of Aberdeen, Aberdeen, AB24 3UE, United Kingdom; 4 INFN Sez. Firenze, via Sansone, 1 - I-50019, Sesto Fiorentino, Italy

**Keywords:** pulse-coupled neural networks, Floquet spectra, splay states

## Abstract

We analytically investigate the stability of *splay states* in the networks of *N* globally pulse-coupled phase-like models of neurons. We develop a perturbative technique which allows determining the Floquet exponents for a generic velocity field and implement the method for a given pulse shape. We find that in the case of discontinuous velocity fields, the Floquet spectrum scales as $1/N^{2}$ and the stability is determined by the sign of the jump at the discontinuity. Altogether, the form of the spectrum depends on the pulse shape, but it is independent of the velocity field.

**PACS:** 05.45.Xt, 84.35.+i, 87.19.lj.

## 1 Introduction

The first objective of (neural) network theory is the identification of asymptotic regimes. Previous research activity led to the discovery of fully- and partially-synchronised states, clusters and splay or asynchronous states in pulse-coupled networks [1-4]. It has also been made clear that ingredients such as disorder (the diversity of neurons and the structure of connections) are very important in determining the asymptotic behaviour, as well as the possible presence of delayed interactions and plasticity [5,6]. However, even if one restricts the analysis to identical, globally-coupled oscillators, there are very few theoretical results and they mostly concern fully-synchronised regime or specific types of neurons (*e.g.* the leaky integrate-and-fire model) [4,7,8]. 

In this paper, we develop a perturbative analysis for the stability of *splay states* (also known as antiphase states [9], ‘ponies on a merry-go-round’ [10], or rotating waves [11]) in ensembles of *N* globally pulse-coupled identical neurons. In a splay state, all the neurons follow the same periodic dynamics and their phases are evenly shifted. Accordingly, the phase, and potential, separation is of order 1/N. Splay states have been identified in experimental measurements performed on electronic circuits [11] and on multimode lasers [12]. Theoretical studies have been devoted to splay states in fully-coupled Ginzburg-Landau equations [13], Josephson arrays [14,15], laser models [16], traffic models [17], unidirectionally delay-coupled Stuart-Landau oscillators [18] and pulse-coupled neuronal networks [2]. In the latter context, splay states have been mainly investigated in leaky-integrate-and-fire (LIF) neurons [2,3,19-21], but some studies have been also devoted to the *θ*-neurons [22] and to more realistic neuronal models [23]. Finally, splay states are important in that they provide the simplest instance of asynchronous behaviour and can be thereby used as a testing ground for the stability of a more general class of dynamical regimes. 

Our model neurons are characterised by a membrane potential *u* that is continuously driven by the velocity field F(u) from the resetting value u=0 toward the threshold u=1 (see the next section for a more precise definition). As threshold and resetting value can be identified with one another and thereby *u* interpreted as a phase, it will be customary to refer to the case F(1)≠F(0) as to that of a discontinuous velocity field. Additionally, we assume that the post-synaptic potential (PSP) has a stereotyped shape, the so-called *α*-pulse, that is characterised by identical rise and decay time 1/α[2]. The linear stability analysis reveals that the eigenvectors are characterised by different spatial frequencies (when moving from the neuron with the smallest to that one with the largest membrane potential). It is therefore convenient to use the frequency *ϕ* (scaled to the average phase separation 1/N) to parametrise the Floquet spectrum. As already discussed in [21], there exist two components, namely short (SWs) and long (LWs) wavelengths. SWs vary on ‘microscopic’ scales, *i.e.* correspond to finite *ϕ* values: they are typically marginally stable in the thermodynamic limit (N→∞). LWs vary on scales of order O(1), *i.e.* correspond to vanishing frequencies: they have been studied in the context of mean-field theory, *i.e.* by analysing a suitable functional equation for the probability distribution of the membrane potential *u*[2,24]. By developing an approach that is valid for arbitrary coupling strength and is perturbative in the inverse system-size 1/N, here we prove that the Floquet spectrum scales as $1/N^{2}$ and is proportional to F(1)−F(0). We are also able to determine the spectral shape and find it to be independent of the structure of the velocity field. The transition from SWs to LWs is signalled by the occurrence of a singularity in the spectrum for the frequency ϕ→0. In the crossover region ϕ=k/N (where *k* is large but small compared to *N*), we show that the exponents remain finite and coincide with those determined in the weak coupling limit by Abbott and Van Vreeswijk [2] with their mean field approach. This result is non-trivial, since it is not *a priori* obvious that the ‘macroscopic’ description discussed in [2] is fully contained in the ‘microscopic’ description derived in this paper, as they refer to two different levels of description. 

More specifically, we first build a suitable event-driven map and expand it in powers of 1/N (*a posteriori*, we have verified that it is necessary to reach the fourth order). Afterwards, an expression of the splay state is determined: this task corresponds to finding a fixed point of the event-driven map in a suitably moving reference frame - analogously to what has previously been done in specific contexts [21,25,26]. In practise this task is carried out by first taking the continuum limit for various orders and then obtaining suitable differential equations. The solutions of such equations show that all finite-size corrections for both the period *T* and the membrane potential vanish up to the third order. Next, the stability analysis is carried out to determine the leading term of the Floquet spectrum. This task involves the introduction of a suitable Ansatz to decompose each eigenvector into the linear superposition of a slow and rapidly oscillating component. The following continuum limit shows that the two components satisfy an ordinary and a differential equation, respectively.

Altogether, the proof of our main result requires determining all terms up to the third order in the 1/N expansion of the splay state solution, while some third-order terms are not necessary for the tangent space analysis. Going beyond discontinuous fields would require extending our analysis to account for higher-order terms and this might not even be sufficient to characterise analytic velocity fields. In fact, previous numerical simulations [25] suggest that the Floquet exponents scale with higher powers of 1/N depends on which derivatives of F(u) are eventually discontinuous. Moreover, it is worth recalling that in the case of a strictly sinusoidal field, the theorem proved by Watanabe and Strogatz [27] implies that N−3 Floquet exponents (N−2 for a splay solution) vanish exactly for any value of *N*.

From the analysis of the SW spectra, one can conclude that the splay state is stable in excitatory (inhibitory) networks whenever F(0)>F(1) (F(0)<F(1)). These conditions can be extended also to the crossover region, where our results coincide with those obtained in [2] (in the limit of a small coupling). Our analytical studies cannot, however, predict the behaviour of the LW component that may be responsible for the emergence of new collective solutions in excitatory networks [3,28]. The overall scenario is partially reminiscent of the stability of synchronous and clustered regimes that is determined by the sign of the first derivative dF/du of the velocity-field averaged on the interval [0,1] (the latter problem has been investigated in excitatory pulse-coupled integrate-and-fire oscillators subject to *δ*-pulses [1,29]). 

Section 2 is devoted to the introduction of the model and to a brief presentation of the main results, including an expression for the leading correction to the period for the LIF model, to provide evidence that it is typically of the fourth order. A general perturbative expression for the map is derived in Section 3, while Section 4 is devoted to deriving the splay state solution up to the third order in 1/N. The main result of the paper is discussed in Section 5, where the Floquet spectra are finally obtained. Section 6 contains some general remarks and a discussion of open problems. The technical details of some lengthy calculations have been confined in the appendices: Appendix A is devoted to the derivation of the splay state solution; Appendix B contains the derivation of the leading term (of order four) of the period *T* for the LIF model; Appendix C is concerned with the linear stability analysis.

## 2 Model and main results

We consider a network of *N* identical neurons (rotators) coupled via a mean-field term. The dynamics of the *i*th neuron writes as

(1) $${\dot{u}}_{i}(t)=F(u_{i})+gE(t)\equiv F_{i}(t),\;i=1,\ldots ,N,$$

where $u_{i}(t)$ represents the membrane potential, E(t) is the forcing field and *g* is the coupling constant. When the membrane potential reaches the threshold value $u_{i}(t)=u_{\mathrm{th}}=1$, a spike is sent to all neurons (see below for the relationship between the single spikes and the global forcing field E(t)) and it is reset to $u_{i}(t)=u_{R}=0$. The resetting procedure is an approximate way to describe the discharge mechanism operating in real neurons. The function *F* represents a velocity field for the isolated neuron and it is assumed to be everywhere positive (thus ensuring that the neurons repetitively fire, since they are supra-threshold), while $F_{i}$ is the velocity field seen by the neuron *i* in the presence of a coupling with other neurons. While we consider both excitatory (g>0) and inhibitory networks (g<0), it is easy to show that ℱ remains always positive to ensure the existence of splay states. For the simple choice

**Fig. 1:**
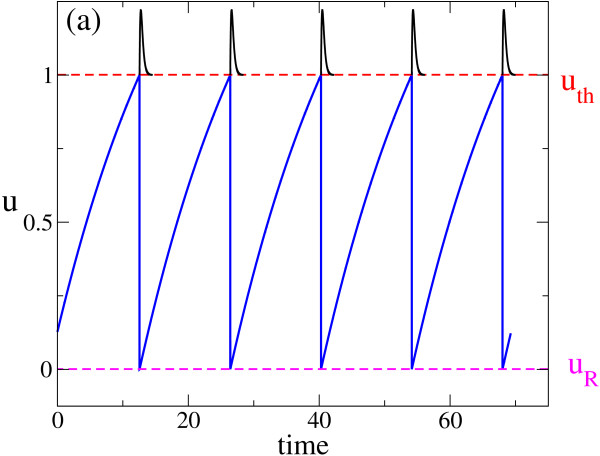
(**a**) Temporal course of the membrane potential for a suprathreshold LIF neuron in the absence of synaptic stimuli. The pulses $E_{s}$ are exactly collocated in correspondence of the firing times. $u_{R}$ represents the reset value and $u_{\mathrm{th}}$ the threshold value. (**b**) ‘Raster plot’ for a splay state in a network of N=100 neurons: neuron indices are reported as a function of firing times. The *dots* correspond to the times when the neurons emit an action potential. Indices are ordered according to the (first) time the neurons have reached the threshold.

(2) F(u)=a−u,

the model reduces to the well-known case of LIF neurons. The evolution of a membrane potential for a LIF suprathreshold neuron (a>1) is reported in Figure 1(a). 

The field *E* is the linear superposition of the pulses emitted in the past when the membrane potential of each single neuron had reached the threshold value. By following Ref. [2], we assume that the shape of a pulse emitted at time t=0 is given by $E_{s}(t)=\frac{\alpha^{2}t}{N}e^{-\alpha t}$, where 1/α is the pulse-width. This is equivalent to saying that the total field evolves according to the equation

(3) $$\ddot{E}(t)+2\alpha \dot{E}(t)+\alpha^{2}E(t)=\frac{\alpha^{2}}{N}\sum_{n|t_{n}<t}\delta (t-t_{n}),$$

where the sum in the rhs represents the source term due to the spikes emitted at times $t_{n}<t$.

It is convenient to transform the continuous-time model into a discrete-time mapping. We do so by integrating the equations of motion from time $t_{n}$ to time $t_{n+1}$ (where $t_{n}$ is the time immediately after the *n*th pulse has been emitted). The resulting map for the field variables reads

(4) $$\begin{array}{rl} E_{n+1} & =[E_{n}+\tau_{n}P_{n}]e^{-\alpha \tau_{n}},\\ P_{n+1} & =P_{n}e^{-\alpha \tau_{n}}+\frac{\alpha^{2}}{N}, \end{array}$$

where $\tau_{n}=t_{n+1}-t_{n}$ is the interspike time interval and, for the sake of simplicity, we have introduced the new variable $P:=\alpha E+\dot{E}$.

In this paper we focus on a specific solution of the network dynamics, namely on *splay states*, which are asynchronous states, where all neurons fire periodically with the period *T* and two successive spike emissions occur at regular intervals $\tau_{n}\equiv T/N$. A typical ‘raster plot’ for this state is reported in Figure 1(b).

In the large-*N* limit, it is natural to consider 1/N as a smallness parameter and thereby to expand the evolution equations in powers of 1/N. In order to perform this expansion, the unique condition to require is the differentiability of the velocity field F(u) in the definition interval ]0,1[. The only exception is represented by the boundaries of the interval where discontinuity is allowed.

The first result of this paper is that under the assumption that the velocity field F(u) is differentiable at least four times, the dependence of the period *T* onto the size *N* is of order $o(1/N^{3})$. In the specific case of LIF neurons, we show in Appendix B that the leading correction *δT* to the infinite size result is indeed of order $O(1/N^{4})$ and, more precisely,

(5) $$\delta T=\frac{K(\alpha )-6}{720}\frac{\left[a(1-e^{-T})-1\right]}{ge^{-T}+a(T+1-e^{-T})-1}\frac{T^{5}}{N^{4}},$$

where K(α) encodes the information on the pulse dynamics (see Eq. (71)). We did not dare to estimate the quartic contribution for generic velocity fields, not only because the algebra would be utterly complicated, but also since our main motivation is to determine the leading contributions in the stability analysis, and it turns out that it is sufficient to determine the splay state up to the third order.

The study of the stability requires determining the Floquet spectrum, *i.e.* the complex eigenvalues of a given periodic orbit of the period *T*. With reference to a system of size *N* and by following [21], the Floquet multipliers can be written as

(6) $$\begin{array}{rl} \mu_{k} & =e^{i\phi_{k}}e^{(\lambda_{k}+i\omega_{k})\tau_{n}},\;\phi_{k}=2\pi k/N,k=1,\ldots ,N-1,\\ \mu_{N} & =e^{(\lambda_{N}+i\omega_{N})\tau_{n}},\;\mu_{N+1}=e^{(\lambda_{N+1}+i\omega_{N+1})\tau_{n}}, \end{array}$$

where $\phi_{k}$ represents a zeroth-order phase, while $\lambda_{k}$ and $\omega_{k}$ are the real and imaginary parts of the Floquet exponents, respectively.

Notice that since the total number of exponents is N+1 (the zero exponent has been removed by taking the Poincaré section), we are going to miss one of them. Furthermore, as shown in [21], the *N*th and (N+1)th exponents are associated to the field evolution and they will be not considered in our analysis.

In the following we prove that the leading term of the spectrum is

(7) $$\lambda_{k}=\frac{g\alpha^{2}}{12}\frac{F(1)-F(0)}{F(1)F(0)}\left(\frac{6}{1-\cos\phi_{k}}-1\right)\frac{1}{N^{2}},\;k=1,\ldots ,N-1,$$

*i.e.* for discontinuous velocity fields, the real part of the spectrum scales as $1/N^{2}$, while the imaginary part is of even higher order.

For continuous fields, it has been numerically observed that the scaling of the spectrum is at least $O(1/N^{4})$[25]. In other words, the shape of the spectrum is universal, apart from a multiplicative factor that vanishes if and only if F(1)=F(0), *i.e.* for true phase rotators where u=0 coincides with u=1.

In the limit $\phi_{k}\to 0$, Eq. (7) is singular and $\lambda_{k}$ seems to diverge. However, one should recall that $\phi_{k}=2\pi k/N$, *i.e.* it is a discrete variable in a finite system. By expanding for k≪N, one finds that

(8) $$\lambda_{k}=\frac{g\alpha^{2}}{4\pi^{2}k^{2}}\frac{F(1)-F(0)}{F(1)F(0)}.$$

This expression, which holds in the limit of (1≪k≪N), can be compared with the results obtained in [2], in the small-coupling limit (|g|≪1), for sufficiently large *k*. The equivalence of the methods has two implications: (i) the crossover component is ‘universal’ as it is valid also for large coupling constants; (ii) the ‘macroscopic’ stability is fully contained in our ‘microscopic’ analysis. In fact, numerical studies reveal a perfect correspondence also for the LW component that is not amenable to an analytic treatment [25,30]. 

From Eqs. (7) and (8), it follows that the stability of the splay state can be inferred, for arbitrary coupling strength, from the sign of F(1)−F(0): in excitatory (inhibitory) networks, the state is stable whenever F(0)>F(1) (F(0)<F(1)). The same result was previously reported in the weak coupling limit in [2].^a^ It is, however, necessary to point out that such condition(s) do not account for instabilities that can arise in the LW component. This is, *e.g.*, the case of the onset of *partial synchronisation* via a supercritical Hopf bifurcation [3,28]. 

## 3 Event-driven map

By following Ref. [25,31], it is convenient to pass from a continuous to a discrete time evolution rule by introducing the event-driven map which connects the network configuration at subsequent spike emissions occurring at time $t_{n}$ and $t_{n+1}$. The membrane-potential value $u_{i}^{-}(t_{n+1})$ just before the emission of the (n+1)st spike can be obtained by formally integrating Eq. (1),

(9) $$\begin{array}{rcl} u_{i}^{-}(t_{n+1})-u_{n,i}(t_{n}) & = & \int_{t_{n}}^{t_{n+1}}\;dtF\left(u_{i}(t)\right)+g\int_{t_{n}}^{t_{n+1}}\;dt\left[E_{n}+(t-t_{n})P_{n}\right]e^{-\alpha (t-t_{n})}\\ & \equiv & A_{1}(u_{i})+A_{2}(E_{n},P_{n}), \end{array}$$

where the minus superscript means that the map construction has not yet been completed. This task is accomplished by ordering the membrane potentials from the largest (j=1) to the smallest (j=N) value and by passing to a comoving frame that advances with the firing neuron, *i.e.* by shifting the neuron index by one unit $u_{n+1,j-1}=u_{j}^{-}(t_{n+1})$, where the first subscript indicates that the variable is determined at time $t_{n+1}$. As a result, the evolution is described by an event-driven map that is composed of Eq. (4) together with the following recursive relation:

(10) $$u_{n+1,j-1}=u_{n,j}+A_{1}(u_{j},\tau_{n})+A_{2}(E_{n},P_{n},\tau_{n}),$$

where $\tau_{n}=t_{n+1}-t_{n}$. The time at which the (n+1)st spike is emitted can be determined implicitly from Eq. (10) by setting j=1 since, by definition of the model, $u_{n,0}\equiv 1$. In this reference frame the splay state corresponds to a fixed point of the map.

Now we perform a perturbative expansion of both terms $A_{1}$ and $A_{2}$ (see Eq. (9)) in powers of $\delta t=t-t_{n}$. This is justified by the smallness of *δt* which is O(1/N). More precisely, the first integral appearing on the rhs of Eq. (9) is solved perturbatively by introducing a polynomial expansion of $u_{i}(t)$ around $t=t_{n}$,

(11) $$u_{j}(t)=u_{n,j}+{\dot{u}}_{n,j}\delta t+\frac{1}{2}{\ddot{u}}_{n,j}\delta t^{2}+\frac{1}{6}{\overset{\ldots }{u}}_{n,j}\delta t^{3}+O\left(\delta t^{4}\right).$$

Explicit expressions for the time derivates of $u_{j}$ can be obtained from Eq. (1) and its time derivatives,

$$\begin{array}{rcl} {\ddot{u}}_{n,j} & = & F^{\prime }(u_{n,j}){\dot{u}}_{n,j}+g{\dot{E}}_{n},\\ {\overset{\ldots }{u}}_{n,j} & = & F^{\prime \prime }(u_{n,j}){\dot{u}}_{n,j}^{2}+F^{\prime }(u_{n,j}){\ddot{u}}_{n,j}+g{\ddot{E}}_{n}, \end{array}$$

where one can further eliminate ${\ddot{E}}_{n}$ with the help of Eq. (3).

By inserting the expansion (11) into the expression of $A_{1}$, expanding the function F(u) around $u_{n,j}$ and performing the trivial integrations, one obtains

(12) $$\begin{array}{rcl} A_{1} & = & F_{n,j}\tau_{n}+F_{n,j}^{\prime }F_{n,j}\frac{\tau_{n}^{2}}{2}+\left\{\left[F_{n,j}^{\prime \prime }F_{n,j}+F_{n,j}^{\prime 2}\right]F_{n,j}+g{\dot{E}}_{n}F_{n,j}^{\prime }\right\}\frac{\tau_{n}^{3}}{6}\\ & & +\{F_{n,j}^{\prime \prime \prime }F_{n,j}^{3}+4F_{n,j}^{\prime }F_{n,j}^{\prime \prime }F_{n,j}^{2}+F_{n,j}^{\prime 3}F_{n,j}\\ & & +g\left[\left(3F_{n,j}^{\prime \prime }F_{n,j}+F_{n,j}^{\prime 2}-\alpha F_{n,j}^{\prime }\right){\dot{E}}_{n}-\alpha F_{n,j}^{\prime }P_{n}\right]\}\frac{\tau_{n}^{4}}{24}+O\left(\tau_{n}^{5}\right), \end{array}$$

where we have introduced the short-hand notation $F_{n,j}$ for $F(u_{n,j})$ (and analogously for ℱ).

In the case of $A_{2}$, it is possible to obtain an exact expression for the integral, which can be then expanded in powers of $\tau_{n}$ as follows:

(13) $$\begin{array}{rcl} A_{2} & = & \frac{g}{\alpha }E_{n}\left(1-e^{-\alpha \tau_{n}}\right)-\frac{g}{\alpha }P_{n}\tau_{n}e^{-\alpha \tau_{n}}+\frac{g}{\alpha^{2}}P_{n}\left(1-e^{-\alpha \tau_{n}}\right)\\ & = & gE_{n}\tau_{n}+g{\dot{E}}_{n}\frac{\tau_{n}^{2}}{2}-g\alpha ({\dot{E}}_{n}+P_{n})\frac{\tau_{n}^{3}}{6}+g\alpha^{2}({\dot{E}}_{n}+2P_{n})\frac{\tau_{n}^{4}}{24}\\ & & +O\left(\tau_{n}^{5}\right). \end{array}$$

By finally assembling Eqs. (10), (12), (13), we obtain a perturbative expression for the evolution rule of the membrane potential,

(14) $$\begin{array}{rcl} u_{n+1,j-1} & = & u_{n,j}+F_{n,j}\tau_{n}+\left[g{\dot{E}}_{n}+F_{n,j}^{\prime }F_{n,j}\right]\frac{\tau_{n}^{2}}{2}\\ & & +\left\{F_{n,j}^{\prime }\left[F_{n,j}^{\prime }F_{n,j}+g{\dot{E}}_{n}\right]+F_{n,j}^{\prime \prime }F_{n,j}^{2}-g\alpha [P_{n}+{\dot{E}}_{n}]\right\}\frac{\tau_{n}^{3}}{6}\\ & & +\{-g\alpha ({\dot{E}}_{n}+P_{n})F_{n,j}^{\prime }+4F_{n,j}^{\prime }F_{n,j}^{\prime \prime }F_{n,j}^{2}\\ & & +F_{n,j}^{\prime 3}F_{n,j}+g\left[\left(3F_{n,j}^{\prime \prime }F_{n,j}+F_{n,j}^{\prime 2}\right){\dot{E}}_{n}+\alpha^{2}({\dot{E}}_{n}+2P_{n})\right]\\ & & +F_{n,j}^{\prime \prime \prime }F_{n,j}^{3}\}\frac{\tau_{n}^{4}}{24}+O\left(\tau_{n}^{5}\right). \end{array}$$

## 4 Splay state solution

The splay state is a fixed point of the event-driven mapping with a constant interspike interval τ=T/N. Since the fixed-point solutions do not depend on the index *n*, they are denoted as

(15) $$E_{n}\equiv \tilde{E},\;P_{n}\equiv \tilde{P},\;u_{n,j}\equiv {\tilde{u}}_{j}.$$

Substituting Eq. (15) into Eq. (4), one obtains

(16) $$\tilde{P}=\frac{\alpha^{2}}{N}\frac{1}{\left(1-e^{-\alpha T/N}\right)},\;\tilde{E}=\frac{T}{N}\frac{\tilde{P}}{\left(e^{\alpha T/N}-1\right)}.$$

By introducing Eq. (16) in Eq. (10) and eliminating the *n* dependence, we obtain a recursive equation for the variable ${\tilde{u}}_{j}$. The fixed-point solution corresponds to the ‘trajectory’ that, starting from ${\tilde{u}}_{N}=0$, ends in ${\tilde{u}}_{0}=1$ and can be found by tuning the ‘parameter’ T/N. The existence of one or more solutions is related to the dependence on *T*. Simple calculations reveal that $A_{2}=g/N$, *i.e.* it is independent of *T*; moreover, since $A_{1}$ is the integral from time 0 to time T/N of a positive defined function (F>0), it is a monotonically increasing function of *T* which vanishes in zero. Accordingly, for any function *F*, the minimal value of $u_{0}$ is *g* (obtained for T=0), while the maximal value is unbounded from above. Therefore, there exists one and only one solution provided that g<1.

The variables $\tilde{E}$ and $\tilde{P}$ depend explicitly on *N*, and it is natural to expect that the period *T* itself varies with *N*. By replacing the expansion of *T* in powers of the smallness parameter 1/N,

(17) $$T=\sum_{h=0}^{4}\frac{T^{\left(h\right)}}{N^{h}}+O\left(\frac{1}{N^{5}}\right),$$

in Eq. (16) we obtain perturbative expressions for $\tilde{P}$, $\tilde{E}$ and $\dot{\tilde{E}}$, namely Eqs. (60), (61), (62) reported in Appendix A. As for the membrane potential, it is necessary to introduce the formal expansion

(18) $${\tilde{u}}_{j}=\sum_{h=0}^{4}\frac{{\tilde{u}}_{j}^{\left(h\right)}}{N^{h}}+O\left(\frac{1}{N^{5}}\right).$$

Substituting the expansions of *T*, ${\tilde{u}}_{j}$, $\tilde{E}$ and $\tilde{P}$ into Eq. (14) (after having dropped the *n* dependence and expanded the ‘*F*’ functions), one obtains an equation for ${\tilde{u}}_{j}^{\left(h\right)}$ and $T^{\left(h\right)}$, namely

(19) $$\sum_{h=0}^{4}\frac{{\tilde{u}}_{j-1}^{\left(h\right)}-{\tilde{u}}_{j}^{\left(h\right)}}{N^{h}}=\sum_{h=1}^{4}\frac{Q^{\left(h\right)}}{N^{h}}+O\left(\frac{1}{N^{5}}\right),$$

where the Q variables are defined in Appendix A. Notice the dependence on $T^{\left(h\right)}$ variables is hidden in Q terms.

In the large-*N* limit, one can introduce the continuous spatial coordinate x=j/N. In practise, this is tantamount to write

(20) $$U^{\left(h\right)}(x=j/N)=\lim_{N\to \infty }{\tilde{u}}_{j}^{\left(h\right)},\;h=0,\ldots ,4.$$

It is important to stress that the event-driven neuronal evolution in the comoving frame implies that U(0)=1, *i.e.* the first neuron will fire at the next step, and U(1)=0, *i.e.* the membrane potential of the last neuron has been just reset to zero. This implies that $U^{\left(0\right)}(0)=1$ and $U^{\left(0\right)}(1)=0$, while $U^{\left(h\right)}(0)=U^{\left(h\right)}(1)=0$ for any h>0.

Furthermore, by expanding $U^{\left(h\right)}(x)$ around x=j/N, one obtains

(21) $$U^{\left(h\right)}(x-1/N)=U^{\left(h\right)}(x)+\sum_{m=1}^{4}\frac{1}{m!}{\left(\frac{-1}{N}\right)}^{m}\frac{d^{m}}{dx^{m}}U^{\left(h\right)}(x)+O\left(\frac{1}{N^{5}}\right).$$

By inserting this expansion into Eq. (19), we obtain an equation that can be effectively split into terms of different order that will be analysed separately. Notice that by retaining terms of order *h*, it is possible to determine the original variables at order h−1.

### 4.1 Zeroth-order approximation

By assembling first-order terms, we obtain the evolution equation for the zeroth-order membrane potential, namely

(22) $$\frac{dU^{\left(0\right)}}{dx}=-g-T^{\left(0\right)}F\left(U^{\left(0\right)}\right).$$

This equation is equal to the evolution equation of the membrane potential for a constant field *E*, with *x* playing the role of (inverse) time. Please notice that up to the first order, $\tilde{E}=1/T^{\left(0\right)}$ (see Eq. (61)). An implicit and formal solution of Eq. (22) is

(23) $$1-x=\int_{0}^{U^{\left(0\right)}}\frac{dv}{g+T^{\left(0\right)}F(v)},$$

where we have imposed the condition $U^{\left(0\right)}(1)=0$. However, there is the second condition to impose, namely $U^{\left(0\right)}(0)=1$. This second condition transforms itself in the equation defining the interspike time interval $T^{\left(0\right)}$, when N→∞ (*i.e.* in the thermodynamic limit)

(24) $$1=\int_{0}^{1}\frac{dU^{\left(0\right)}}{g+T^{\left(0\right)}F(U^{\left(0\right)})}.$$

This result is, so far, quite standard and could have been easily obtained by just assuming a constant field *E* in Eq. (1). If we introduce the formal relation $F^{\prime }[U^{\left(0\right)}(x)]=\frac{dF(U^{\left(0\right)})}{dU^{\left(0\right)}}$ in Eq. (22), we obtain

(25) $$\frac{dF(U^{\left(0\right)})}{g+T^{\left(0\right)}F(U^{\left(0\right)})}=-F^{\prime }\left[U^{\left(0\right)}(x)\right]\;dx,$$

which can be easily integrated

(26) $$\int_{F(U^{\left(0\right)}(0))}^{F(U^{\left(0\right)}(1))}\frac{dF(U^{\left(0\right)})}{g+T^{\left(0\right)}F(U^{\left(0\right)})}=-\int_{0}^{1}F^{\prime }\left[U^{\left(0\right)}(x)\right]\;dx,$$

giving the following relation (already derived in [28], by following a different approach)

(27) $$\frac{e^{-T^{\left(0\right)}H(0)}}{F(U(0))}=\frac{e^{-T^{\left(0\right)}H(1)}}{F(U(1))},$$

where, for later convenience, we have introduced

(28) $$H(x)=\int_{0}^{x}F^{\prime }\left[U^{\left(0\right)}(y)\right]\;dy,$$

and where, for the sake of simplicity, the prime denotes derivative with respect to the variable $U^{\left(0\right)}$ and the dependence of *F* and $F^{\prime }$ on $U^{\left(0\right)}$ has been dropped.

### 4.2 First-order approximation

By collecting the terms of order $1/N^{2}$, one obtains

(29) $$\frac{dU^{\left(1\right)}}{dx}=-T^{\left(0\right)}F^{\prime }U^{\left(1\right)}+\frac{1}{2}\frac{d^{2}U^{\left(0\right)}}{dx^{2}}-FT^{\left(1\right)}-\frac{1}{2}{\left(T^{\left(0\right)}\right)}^{2}F^{\prime }F-\frac{g}{2}T^{\left(0\right)}F^{\prime }.$$

An explicit expression for the second derivative of $U^{\left(0\right)}(x)$ appearing in Eq. (29) can be computed by deriving Eq. (22) with respect to *x*. This allows rewriting Eq. (29) in a simplified form, namely

(30) $$\frac{dU^{\left(1\right)}(x)}{dx}=-U^{\left(1\right)}T^{\left(0\right)}F^{\prime }-T^{\left(1\right)}F.$$

By imposing $U^{\left(1\right)}(1)=0$, one obtains the general solution of Eq. (30),

(31) $$U^{\left(1\right)}(x)=\int_{x}^{1}duT^{\left(1\right)}F\left[U^{\left(0\right)}(u)\right]\exp\left[T^{\left(0\right)}\left(H(x)-H(u)\right)\right],$$

where H(x) is defined by Eq. (28). The further condition to be satisfied, $U^{\left(1\right)}(0)=0$, implies $T^{\left(1\right)}=0$ and thereby we have $U^{\left(1\right)}(x)\equiv 0$, *i.e.* first-order corrections vanish both for the period and the membrane potential.

### 4.3 Second-order approximation

Second-order corrections can be estimated by assembling terms of order $1/N^{3}$ and by imposing the previously determined conditions $T^{\left(1\right)}=0$ and $U^{\left(1\right)}(x)=0$,

$$\begin{array}{rcl} \frac{dU^{\left(2\right)}}{dx} & = & -T^{\left(0\right)}F^{\prime }U^{\left(2\right)}-\frac{1}{6}\frac{d^{3}U^{\left(0\right)}}{dx^{3}}-FT^{\left(2\right)}-\frac{g^{2}}{6}T^{\left(0\right)}F^{\prime \prime }\\ & & -\frac{g}{6}{\left(T^{\left(0\right)}\right)}^{2}\left[2FF^{\prime \prime }+F^{\prime 2}\right]-\frac{{\left(T^{\left(0\right)}\right)}^{3}}{6}\left[F^{\prime \prime }F^{2}+F^{\prime 2}F\right]. \end{array}$$

Once evaluated $d^{3}U^{\left(0\right)}/dx^{3}$ from Eq. (22), the above ODE reduces to

(32) $$\frac{dU^{\left(2\right)}}{dx}=-U^{\left(2\right)}T^{\left(0\right)}F^{\prime }-T^{\left(2\right)}F,$$

which has the same structure as Eq. (30). Since one has also to impose the same boundary conditions as for the first order, namely $U^{\left(2\right)}(0)=U^{\left(2\right)}(1)=0$, we can conclude that $T^{\left(2\right)}=0$ and, consequently, $U^{\left(2\right)}(x)\equiv 0$. Therefore, second-order corrections are absent too.

### 4.4 Third-order approximation

By assembling terms of order $1/N^{4}$, once imposed that first- and second-order corrections vanish, one obtains

(33) $$\begin{array}{rcl} \frac{dU^{\left(3\right)}}{dx} & = & -T^{\left(0\right)}F^{\prime }U^{\left(3\right)}+\frac{1}{24}\frac{d^{4}U^{\left(0\right)}}{dx^{4}}-FT^{\left(3\right)}\\ & & -\frac{g^{3}}{24}T^{\left(0\right)}F^{\prime \prime \prime }-\frac{g^{2}}{6}{\left(T^{\left(0\right)}\right)}^{2}F^{\prime }F^{\prime \prime }\\ & & -\frac{g^{2}}{8}{\left(T^{\left(0\right)}\right)}^{2}FF^{\prime \prime \prime }-\frac{g}{24}{\left(T^{\left(0\right)}\right)}^{3}\left[F^{\prime 3}+8FF^{\prime }F^{\prime \prime }+3gF^{2}F^{\prime \prime \prime }\right]\\ & & -\frac{{\left(T^{\left(0\right)}\right)}^{4}}{24}FF^{\prime }\left[F^{\prime 2}+4FF^{\prime \prime }+F^{3}F^{\prime \prime \prime }\right]. \end{array}$$

By replacing $d^{4}U^{\left(0\right)}/dx^{4}$ with its expression derived from Eq. (22), Eq. (33) takes the same form as in the two previous examined cases, namely

(34) $$\frac{dU^{\left(3\right)}}{dx}=-U^{\left(3\right)}T^{\left(0\right)}F^{\prime }-T^{\left(3\right)}F.$$

Therefore, we can safely conclude that third-order terms vanish too.

The LIF model can be solved exactly for any value of *N*, starting from the asymptotic value (N→∞). As shown in Appendix B, it turns out that the leading corrections are of the fourth order for both the period *T* and the membrane potential.

## 5 Linear stability analysis

The fixed-point analysis has revealed that the finite-size corrections to the stationary solutions are of order $o(1/N^{3})$. Since such deviations do not affect the leading terms of the linear stability analysis (as it can be verified *a posteriori*) they will be simply neglected. Therefore, for the sake of simplicity, from now on $T^{\left(0\right)}$ and ${\tilde{u}}_{j}^{\left(0\right)}$ will be simply referred to as *T* and ${\tilde{u}}_{j}$.

The evolution rule in the tangent space is obtained by differentiating Eq. (4) and then by expanding in powers of *τ* (this is equivalent to expanding in powers of 1/N, as the dependence of *τ* on *N* would only generate higher-order terms),

(35) $$\delta P_{n+1}=Z^{\left(1\right)}\delta P_{n}+\tilde{P}Z^{\left(2\right)}\delta \tau_{n},$$

(36) $$\delta E_{n+1}=Z^{\left(1\right)}\delta E_{n}+Z^{\left(3\right)}\delta P_{n}+\left[\tilde{P}Z^{\left(4\right)}-\alpha \tilde{E}Z^{\left(1\right)}\right]\delta \tau_{n},$$

where the Z variables are polynomials of *τ* defined in Appendix C.1.

By further differentiating Eq. (14) around the fixed-point solution, one obtains

(37) $$\delta u_{n+1,j-1}=W^{\left(1\right)}\delta u_{n,j}+W^{\left(2\right)}\delta E_{n}+W^{\left(3\right)}\delta {\dot{E}}_{n}+W^{\left(4\right)}\delta \tau_{n}.$$

Finally, $\delta \tau_{n}$ can be determined by differentiating Eq. (14) for j=1

(38) $$\delta \tau_{n}=-\frac{\delta E_{n}}{{\bar{F}}_{1}}W^{\left(5\right)}-\frac{\delta P_{n}}{{\bar{F}}_{1}}W^{\left(6\right)}-\frac{\delta u_{n,1}}{{\bar{F}}_{1}}W^{\left(7\right)},$$

where the auxiliary W variables are defined in Appendix C.1.

As usual, the eigenvalue problem can be solved by introducing the Ansatz,

(39) $$\delta u_{n,j}=\mu_{k}^{n}\delta u_{j},\;\;\delta P_{n}=\mu_{k}^{n}\delta P,\;\;\delta E_{n}=\mu_{k}^{n}\delta E,\;\;\delta \tau_{n}=\mu_{k}^{n}\delta \tau ,$$

where $\mu_{k}$ labels the eigenvalues, which must also be expanded as

(40) $$\mu_{k}=e^{i\phi_{k}}e^{(\lambda_{k}+i\omega_{k})T/N}=e^{i\phi_{k}}\left(1+\sum_{h=1}^{3}\frac{\Gamma^{\left(h\right)}}{N^{h}}+O\left(\frac{1}{N^{4}}\right)\right),$$

where $\Gamma^{\left(h\right)}$ is, in principle, a complex number and, for the sake of simplicity, we have dropped its dependence on *k*. Finally, as already shown at the zeroth order, the eigenvalues correspond to a pure rotation (specified by $\phi_{k}$) with no expansion or contraction, *i.e.*$\Gamma^{\left(0\right)}=0$.

By inserting the above Ansätze in the map expression (35), (36), (37), (38), one obtains, after eliminating *δP*, *δE* and *δτ*, a closed equation for $\delta u_{j}$,

(41) $$\begin{array}{rcl} & & e^{i\phi_{k}}\left(1+\frac{\Gamma^{\left(1\right)}}{N}+\frac{\Gamma^{\left(2\right)}}{N^{2}}+\frac{\Gamma^{\left(3\right)}}{N^{3}}\right)\delta u_{j-1}\\ & & \;=\left\{1+{\bar{F}}_{j}^{\prime }\frac{T}{N}+\left[\;{\bar{F}}_{j}^{\prime \prime }{\bar{F}}_{j}+{\bar{F}}_{j}^{\prime 2}\right]\frac{T^{2}}{2N^{2}}+\left[\;{\bar{F}}_{j}^{\prime \prime \prime }{\bar{F}}_{j}^{2}+4{\bar{F}}_{j}^{\prime }{\bar{F}}_{j}^{\prime \prime }{\bar{F}}_{j}+{\bar{F}}_{j}^{\prime 3}\right]\frac{T^{3}}{6N^{3}}\right\}\delta u_{j}\\ & & \;\;-\{{\bar{F}}_{j}+{\bar{F}}_{j}^{\prime }{\bar{F}}_{j}\frac{T}{N}+[\frac{{\bar{F}}_{j}^{\prime \prime }{\bar{F}}_{j}^{2}}{2}+\frac{{\bar{F}}_{j}^{\prime 2}}{2}{\bar{F}}_{j}\\ & & \;\;+\frac{g}{T}\alpha^{2}\frac{e^{2i\phi_{k}}+10e^{i\phi_{k}}+1}{12{\left(e^{i\phi_{k}}-1\right)}^{2}}\left(\frac{{\bar{F}}_{j}}{{\bar{F}}_{1}}-1\right)]\frac{T^{2}}{N^{2}}\\ & & \;\;+[\frac{{\bar{F}}_{j}^{\prime 3}}{6}{\bar{F}}_{j}+\frac{2}{3}{\bar{F}}_{j}^{\prime }{\bar{F}}_{j}^{\prime \prime }{\bar{F}}_{j}^{2}+\frac{{\bar{F}}_{j}^{\prime \prime \prime }}{6}{\bar{F}}_{j}^{3}+\frac{g\alpha^{2}}{3T^{2}}\Gamma^{\left(1\right)}\frac{2e^{2i\phi_{k}}-3e^{i\phi_{k}}}{{\left(e^{i\phi_{k}}-1\right)}^{3}}\left(\frac{{\bar{F}}_{j}}{{\bar{F}}_{1}}-1\right)\\ & & \;\;+\frac{g}{T}\alpha^{2}\frac{e^{2i\phi_{k}}+10e^{i\phi_{k}}+1}{12{\left(e^{i\phi_{k}}-1\right)}^{2}}\frac{{\bar{F}}_{j}}{{\bar{F}}_{1}}\left(\;{\bar{F}}_{j}^{\prime }-{\bar{F}}_{1}^{\prime }\right)\\ & & \;\;+\frac{g\alpha^{2}}{T}\frac{5e^{i\phi_{k}}+1}{12{\left(e^{i\phi_{k}}-1\right)}^{2}}\left({\bar{F}}_{1}^{\prime }\frac{{\bar{F}}_{j}}{{\bar{F}}_{1}}-{\bar{F}}_{j}^{\prime }\right)\\ & & \;\;+\frac{g\alpha^{3}}{T}\frac{e^{i\phi_{k}}(e^{i\phi_{k}}+1)}{{\left(e^{i\phi_{k}}-1\right)}^{3}}\left(1-\frac{{\bar{F}}_{j}}{{\bar{F}}_{1}}\right)]\frac{T^{3}}{N^{3}}\}\frac{\delta u_{1}}{{\bar{F}}_{1}}, \end{array}$$

that is the object of our investigation. The overline means that the function is evaluated in ${\tilde{u}}_{j}^{\left(0\right)}$, corresponding to the infinite *N* limit.

### 5.1 Continuum limit

Similarly to the splay state estimation, it is convenient to take the continuum limit. However, at variance with the previous case, now one should take into account also the presence of fast scales associated to the ‘spatial’ dependence of $\phi_{k}$.

Therefore, the correct Ansatz is slightly more complicated, and we have to separate slowly and rapidly oscillating terms,

(42) $$\delta u_{j}=\pi_{j}+\vartheta_{j}e^{i\phi_{k}j},$$

where the complex exponential term accounts for the fast oscillations of the eigenvectors, while

(43) $$\pi_{j}=\sum_{h=0}^{3}\frac{\pi_{j}^{\left(h\right)}}{N^{h}}+O\left(\frac{1}{N^{4}}\right),\;\;\vartheta_{j}=\sum_{h=0}^{3}\frac{\vartheta_{j}^{\left(h\right)}}{N^{h}}+O\left(\frac{1}{N^{4}}\right)$$

are slowly varying variables. On the one hand, the existence of the slow component $\pi_{j}$ follows from the analogy with the real-space evolution. On the other hand, the presence of the rapidly oscillating terms $e^{i\phi_{k}j}$, first noticed in Ref. [2] in the uncoupled limit, suggests the presence of the second slow field, namely $\vartheta_{j}$. Anyway, the correctness and uniqueness of Ansatz (42) is ensured *a posteriori* by the consistency of the equations that are obtained for the various perturbation orders.

Next, we can finally introduce the continuous variable x=j/N, as previously done in a real space (see Eq. (20)),

(44) $$\Pi_{j}^{\left(h\right)}\left(x=\frac{j}{N}\right)=\pi_{j}^{\left(h\right)},\;\;\Theta_{j}^{\left(h\right)}\left(x=\frac{j}{N}\right)=\vartheta_{j}^{\left(h\right)},$$

where h=0,…,3. This allows expanding $\delta u_{j-1}=\pi_{j-1}+\vartheta_{j-1}e^{i\phi_{k}(j-1)}$ around x=j/N, similarly to what has been done in Eq. (21). At variance with the computation of the fixed point, now there are also terms like U(1/N) and δU(1/N), whose computation requires a similar expansion, but around x=0. By incorporating all the expansion terms within Eq. (41), we have finally an equation, where terms of different orders are naturally separated from one another. The calculations are summarised in Appendix C, and the final equation is (86). By separately treating the different orders, we obtain differential and ordinary equations for the Θ and Π variables. It turns out that it is necessary to consider in parallel different orders in the fast and slow terms to obtain Θ and Π to the same order. As a consequence, we will see that it is sufficient to expand δU(1/N) up to order $O(1/N^{3})$.

### 5.2 Zeroth-order approximation

By assembling terms of order O(1/N) in Eq. (86), multiplied by the fast oscillating factor $e^{i\phi_{k}j}$, we obtain a first-order linear differential equation for $\Theta^{\left(0\right)}$, namely

(45) $$\frac{d\Theta^{\left(0\right)}}{dx}=-\Theta^{\left(0\right)}\left(TF^{\prime }\left(U(x)\right)-\Gamma^{\left(1\right)}\right),$$

where $\Gamma^{\left(1\right)}$ is the first-order correction to the Floquet exponent which should be determined. It is important to remind that the prime denotes derivative with respect to the variable $U^{\left(0\right)}$, which has been simply redefined *U*, as previously mentioned. The solution is

(46) $$\Theta^{\left(0\right)}(x)=K^{\left(0\right)}\exp\left[\Gamma^{\left(1\right)}x-TH(x)\right],$$

where we made use of the definition (28) and $K^{\left(0\right)}$ is a suitable integration constant.

By assembling now the slow terms of the zeroth order and reminding the definition of F(U(x)), we find the following algebraic equation:

(47) $$\Pi^{\left(0\right)}(x)\left(e^{i\phi_{k}}-1\right)=-\left[e^{i\phi_{k}}\Theta^{\left(0\right)}(0)+\Pi^{\left(0\right)}(0)\right]\frac{F(U(x))}{F(U(0))}.$$

With the help of Eq. (46), we obtain

$$\begin{array}{rcl} \Pi^{\left(0\right)}(0) & = & -\Theta^{\left(0\right)}(0)=-K^{\left(0\right)}e^{-TH(0)},\\ \Pi^{\left(0\right)}(x) & = & -K^{\left(0\right)}e^{-TH(0)}\frac{F(U(x))}{F(U(0))}. \end{array}$$

We can now impose the boundary condition $\delta U^{\left(0\right)}(x=1)=\Theta^{\left(0\right)}(1)+\Pi^{\left(0\right)}(1)=0$. This implies that

(48) $$\frac{e^{-TH(1)+\Gamma^{\left(1\right)}}}{F(U(1))}=\frac{e^{-TH(0)}}{F(U(0))}.$$

From Eq. (27), we can conclude that $\Gamma^{\left(1\right)}=2\pi im$, for 0≤m<N (values outside this range give identical solutions). Since the vector $\delta u_{j}$ was assumed to be characterised by the phase $j\phi_{j}$ (see Eq. (42)), the phase factor $\Gamma^{\left(1\right)}=2\pi im$ would imply (through Eq. (46)) that $\phi_{k}$ has to be shifted by some amount, contrary to the initial assumption. Accordingly, the only solution consistent with the original Ansatz is m=0, *i.e.*$\Gamma^{\left(1\right)}=0$, and from Eq. (46),

(49) $$\Theta^{\left(0\right)}(x)=K^{\left(0\right)}e^{-TH(x)},$$

*i.e.* the eigenvectors are independent of the phase $\phi_{k}$ and equal to one another. In other words, the degeneracy has not been lifted.

### 5.3 First-order approximation

By assembling the fast terms of order $1/N^{2}$ and by setting $\Gamma^{\left(1\right)}=0$, we find that $\Theta^{\left(1\right)}$ satisfies the following first order differential equation:

(50) $$\frac{d\Theta^{\left(1\right)}}{dx}=\Gamma^{\left(2\right)}\Theta^{\left(0\right)}-\Theta^{\left(1\right)}TF^{\prime }\left(U(x)\right),$$

whose solution is

(51) $$\Theta^{\left(1\right)}(x)=\left(\Gamma^{\left(2\right)}K^{\left(0\right)}x+K^{\left(1\right)}\right)e^{-TH(x)},$$

where $K^{\left(1\right)}$ is an integration constant associated with the solution of the previous equation.

By collecting the slow terms of order 1/N in Eq. (86), one obtains the algebraic equation

(52) $$\Pi^{\left(1\right)}(x)\left(e^{i\phi_{k}}-1\right)=-\left[e^{i\phi_{k}}\Theta^{\left(1\right)}(0)+\Pi^{\left(1\right)}(0)\right]\frac{F(U(x))}{F(U(0))},$$

whose solution is

$$\begin{array}{rcl} \Pi^{\left(1\right)}(0) & = & -\Theta^{\left(1\right)}(0)=-K^{\left(1\right)}e^{-TH(0)},\\ \Pi^{\left(1\right)}(x) & = & -K^{\left(1\right)}e^{-TH(0)}\frac{F(U(x))}{F(U(0))}. \end{array}$$

By imposing the boundary condition $\delta U^{\left(1\right)}(x=1)=\Theta^{\left(1\right)}(1)+\Pi^{\left(1\right)}(1)=0$, it is possible to evaluate $\Gamma^{\left(2\right)}$,

(53) $$\begin{array}{rcl} & & \Theta^{\left(1\right)}(1)+\Pi^{\left(1\right)}(1)\\ & & \;=\left(K^{\left(0\right)}\Gamma^{\left(2\right)}+K^{\left(1\right)}\right)e^{-TH(1)}-K^{\left(1\right)}e^{-TH(0)}\frac{F(U(1))}{F(U(0))}=0. \end{array}$$

Again from Eq. (27) and using the same argument as in the previous section, we find that $\Gamma^{\left(2\right)}=0$ and thereby (from Eq. (51))

(54) $$\Theta^{\left(1\right)}(x)=K^{\left(1\right)}e^{-TH(x)}.$$

Altogether, we can conclude that the second-order correction to the Floquet exponent vanishes as well, and one cannot lift the degeneracy among the eigenvectors.

### 5.4 Second-order approximation

By assembling fast terms of order $1/N^{3}$ appearing in Eq. (86) and by setting $\Gamma^{\left(1\right)}=\Gamma^{\left(2\right)}=0$, the following first-order differential equation for $\Theta^{\left(2\right)}$ can be derived:

(55) $$\frac{d\Theta^{\left(2\right)}}{dx}=\Gamma^{\left(3\right)}\Theta^{\left(0\right)}+\Theta^{\left(2\right)}TF^{\prime }\left(U(x)\right),$$

whose solution is

(56) $$\Theta^{\left(2\right)}(x)=\left(\Gamma^{\left(3\right)}K^{\left(0\right)}x+K^{\left(2\right)}\right)e^{-TH(x)},$$

where $K^{\left(2\right)}$ is an integration constant associated with the solution of the previous differential equation.

Furthermore, by collecting the slow terms of order $1/N^{2}$, we obtain the algebraic equation

$$\begin{array}{rcl} \Pi^{\left(2\right)}(x)\left(e^{i\phi_{k}}-1\right) & = & g\alpha^{2}T\Theta^{\left(0\right)}(0)\frac{e^{2i\phi_{k}}+10e^{i\phi_{k}}+1}{12(e^{i\phi_{k}}-1)}\frac{F(U(0))-F(U(x))}{{\left[F(U(0))\right]}^{2}}\\ & & -\left[e^{i\phi_{k}}\Theta^{\left(2\right)}(0)+\Pi^{\left(2\right)}(0)\right]\frac{F(U(x))}{F(U(0))}. \end{array}$$

By imposing that the above equation is satisfied for x=0, it reduces to

$$\begin{array}{rcl} \Pi^{\left(2\right)}(0) & = & -\Theta^{\left(2\right)}(0)=-K^{\left(2\right)}e^{-TH(0)},\\ \Pi^{\left(2\right)}(x) & = & g\alpha^{2}T\Theta^{\left(0\right)}(0)\frac{e^{2i\phi_{k}}+10e^{i\phi_{k}}+1}{12{\left(e^{i\phi_{k}}-1\right)}^{2}}\frac{F(U(0))-F(U(x))}{{\left[F(U(0))\right]}^{2}}\\ & & -\Theta^{\left(2\right)}(0)\frac{F(U(x))}{F(U(0))}. \end{array}$$

Finally, by imposing the boundary condition $\delta U^{\left(2\right)}(x=1)=\Theta^{\left(2\right)}(1)+\Pi^{\left(2\right)}(1)=0$, it is possible to determine $\Gamma^{\left(3\right)}$,

(57) $$\Gamma^{\left(3\right)}=\frac{g\alpha^{2}}{12}T\frac{F(U(0))-F(U(1))}{F(U(0))F(U(1))}\left(\frac{6}{1-\cos\phi_{k}}-1\right).$$

Accordingly, $\Gamma^{\left(3\right)}$ is real and depends on the difference between F(U(x=1))≡F(0) and F(U(x=0))≡F(1), confirming the numerical findings in [25]. Therefore, the imaginary terms $\omega_{k}$ are smaller than $1/N^{2}$.

In the specific example of a leaky integrate-and-fire neuron, the expression for $\Gamma^{\left(3\right)}$ reduces to

(58) $$\Gamma^{\left(3\right)}=\frac{g\alpha^{2}}{12}T\left(2-e^{T}-e^{-T}\right)\left(\frac{6}{1-\cos\phi_{k}}-1\right),$$

since, by using the equations that characterise LIF neurons, the following relation holds:

(59) $$\frac{F(U(1))-F(U(0))}{F(U(1))F(U(0))}=\frac{1}{{\left(a+\frac{g}{T}\right)}^{2}e^{-T}}=\frac{1}{{\left(\frac{1}{1-e^{-T}}\right)}^{2}e^{-T}}=\left(e^{T}+e^{-T}-2\right).$$

All in all, Eq. (57) generalises the expression found for the LIF model Eq. (58) [25].^b^

## 6 Conclusions

We have derived analytically the short-wavelength component of the Floquet spectrum of the splay solution in a fully-coupled network composed of *generic* suprathreshold pulse-coupled phase-like neurons in the large-*N* limit. Our analysis has revealed that, for discontinuous velocity fields, the spectrum scales as $1/N^{2}$ and the stability is controlled by the sign of the difference between the velocity at reset and at threshold. The shape of the spectrum is otherwise independent of the velocity field. It would be interesting to investigate the role of the pulse shape as well. As long it follows from a linear evolution equation, such as Eq. (3), it is indeed possible to replicate the analysis carried out in this paper. Numerical studies suggest that the scaling behaviour is truly universal, while the shape of the Floquet spectrum depends on the pulse shape [30]. It would be interesting to discover whether and which pulse shapes may give rise to SW instabilities. 

Networks of LIF neurons coupled via *δ*-like pulses are characterised by a finite (in)stability of the whole spectrum [21]. The difference with the case of *α*-pulse is so strong that it cannot be reconciled even by taking the limit α→∞ (zero pulse-width), indicating that the limits N→∞ and zero pulse-width do not commute [21]. This reveals that even the development of general stability theory of the *simple* splay states requires some further progress.

Finally, notice that although our analytical approach is able to cover the entire SW component and the crossover region, it does not cover the truly long wavelengths which require going beyond a perturbative approach.

## Appendix A: Fixed-point expansion (general case)

The 1/N expansion of the exact expressions (16) for $\tilde{P}$ and $\tilde{E}$ leads to

(60) $$\begin{array}{rcl} \tilde{P} & = & \frac{\alpha }{T^{\left(0\right)}}+\left[\frac{\alpha }{2}-\frac{T^{\left(1\right)}}{{T^{\left(0\right)}}^{2}}\right]\frac{\alpha }{N}+\left[\frac{\alpha^{2}T^{\left(0\right)}}{12}-\frac{T^{\left(2\right)}}{{T^{\left(0\right)}}^{2}}+\frac{{T^{\left(1\right)}}^{2}}{{T^{\left(0\right)}}^{2}}\right]\frac{\alpha }{N^{2}}\\ & & +\left[\frac{\alpha^{2}T^{\left(1\right)}}{12}-\frac{T^{\left(3\right)}}{{T^{\left(0\right)}}^{2}}+2\frac{T^{\left(1\right)}T^{\left(2\right)}}{{T^{\left(0\right)}}^{3}}-\frac{{T^{\left(1\right)}}^{3}}{{T^{\left(0\right)}}^{4}}\right]\frac{\alpha }{N^{3}}+O\left(\frac{1}{N^{4}}\right), \end{array}$$

(61) $$\begin{array}{rcl} \tilde{E} & = & \frac{1}{T^{\left(0\right)}}-\frac{T^{\left(1\right)}}{{T^{\left(0\right)}}^{2}N}+\left[-\frac{\alpha^{2}T^{\left(0\right)}}{12}-\frac{T^{\left(2\right)}}{{T^{\left(0\right)}}^{2}}+\frac{{T^{\left(1\right)}}^{2}}{{T^{\left(0\right)}}^{3}}\right]\frac{1}{N^{2}}\\ & & +\left[-\frac{\alpha^{2}}{12}T^{\left(1\right)}-\frac{T^{\left(3\right)}}{{T^{\left(0\right)}}^{2}}+2\frac{T^{\left(1\right)}T^{\left(2\right)}}{{T^{\left(0\right)}}^{3}}-\frac{{T^{\left(1\right)}}^{3}}{{T^{\left(0\right)}}^{4}}\right]\frac{1}{N^{3}}+O\left(\frac{1}{N^{4}}\right), \end{array}$$

(62) $$\dot{\tilde{E}}=\frac{\alpha^{2}}{2N}+\frac{\alpha^{2}T^{\left(0\right)}}{6N^{2}}+\frac{\alpha^{3}T^{\left(1\right)}}{6N^{3}}+O\left(\frac{1}{N^{4}}\right),$$

where we have reported also the expansion of $\dot{\tilde{E}}$ that is necessary to pass from expression (14) to (19). Please notice that while the membrane potentials and the period are expanded up to $O(1/N^{4})$, as in (18) and (17), here we limit the expansion to $O(1/N^{3})$ terms, since the field variables appearing in the event-driven map are integrated over an interspike-interval (see (9)).

To proceed further, we need also to introduce the expansions of the velocity field and of its derivatives,

$$\begin{array}{rcl} F({\tilde{u}}_{j}) & = & {\bar{F}}_{j}+{\bar{F}}_{j}^{\prime }\frac{{\tilde{u}}_{j}^{\left(1\right)}}{N}+{\bar{F}}_{j}^{\prime }\frac{{\tilde{u}}_{j}^{\left(2\right)}}{N^{2}}+{\bar{F}}_{j}^{\prime }\frac{{\tilde{u}}_{j}^{\left(3\right)}}{N^{3}}\\ & & +{\bar{F}}_{j}^{\prime \prime }\frac{{\left[{\tilde{u}}_{j}^{\left(1\right)}\right]}^{2}}{2N^{2}}+{\bar{F}}_{j}^{\prime \prime }\frac{{\tilde{u}}_{j}^{\left(1\right)}{\tilde{u}}_{j}^{\left(2\right)}}{N^{3}}+{\bar{F}}_{j}^{\prime \prime \prime }\frac{{\left[{\tilde{u}}_{j}^{\left(1\right)}\right]}^{3}}{6N^{3}}+O\left(\frac{1}{N^{4}}\right),\\ F^{\prime }({\tilde{u}}_{j}) & = & {\bar{F}}_{j}^{\prime }+{\bar{F}}_{j}^{\prime \prime }\frac{{\tilde{u}}_{j}^{\left(1\right)}}{N}+{\bar{F}}_{j}^{\prime \prime }\frac{{\tilde{u}}_{j}^{\left(2\right)}}{N^{2}}+{\bar{F}}_{j}^{\prime \prime \prime }\frac{{\left[{\tilde{u}}_{j}^{\left(1\right)}\right]}^{2}}{2N^{2}}+O\left(\frac{1}{N^{3}}\right),\\ F^{\prime \prime }({\tilde{u}}_{j}) & = & {\bar{F}}_{j}^{\prime \prime }+{\bar{F}}_{j}^{\prime \prime \prime }\frac{{\tilde{u}}_{j}^{\left(1\right)}}{N}+O\left(\frac{1}{N^{2}}\right), \end{array}$$

where the overline means that the function is computed in $\tilde{U}(x=j/N)$, which corresponds to the infinite *N* limit.

By replacing the membrane potentials, the period, the self-consistent fields and the velocity field with their expansions, the event-driven map (14) can be formally rewritten for the splay state as (19) with the introduction of the following auxiliary variables:

(63) $$Q^{\left(1\right)}=g+T^{\left(0\right)}{\bar{F}}_{j},$$

(64) $$Q^{\left(2\right)}=T^{\left(1\right)}{\bar{F}}_{j}+\left[{\tilde{u}}_{j}^{\left(1\right)}+\frac{g}{2}+\frac{{\bar{F}}_{j}}{2}T^{\left(0\right)}\right]{\bar{F}}_{j}^{\prime }T^{\left(0\right)},$$

(65) $$\begin{array}{rcl} Q^{\left(3\right)} & = & [{\bar{F}}_{j}^{\prime }{\tilde{u}}_{j}^{\left(2\right)}+\frac{{\bar{F}}_{j}^{\prime \prime }}{2}{\left[{\tilde{u}}_{j}^{\left(1\right)}\right]}^{2}+g\frac{{\bar{F}}_{j}^{\prime \prime }}{2}{\tilde{u}}_{j}^{\left(1\right)}+\frac{g^{2}}{6}{\bar{F}}_{j}^{\prime \prime }\\ & & +\left(\frac{2g}{3}{\bar{F}}_{j}^{\prime \prime }{\bar{F}}_{j}+{\bar{F}}_{j}^{\prime 2}{\tilde{u}}_{j}^{\left(1\right)}+{\bar{F}}_{j}^{\prime \prime }{\bar{F}}_{j}{\tilde{u}}_{j}^{\left(1\right)}+\frac{g}{3}{\bar{F}}_{j}^{\prime 2}\right)\frac{T^{\left(0\right)}}{2}\\ & & +\left(\;{\bar{F}}_{j}^{\prime \prime }{\bar{F}}_{j}+{\bar{F}}_{j}^{\prime 2}\right)\frac{{T^{\left(0\right)}}^{2}{\bar{F}}_{j}}{6}]T^{\left(0\right)}\\ & & +\left[{\tilde{u}}_{j}^{\left(1\right)}+\frac{g}{2}+{\bar{F}}_{j}T^{\left(0\right)}\right]T^{\left(1\right)}{\bar{F}}_{j}^{\prime }+T^{\left(2\right)}{\bar{F}}_{j}, \end{array}$$

(66) $$\begin{array}{rcl} Q^{\left(4\right)} & = & [{\bar{F}}_{j}^{\prime }{\tilde{u}}_{j}^{\left(3\right)}+\left({\tilde{u}}_{j}^{\left(1\right)}+\frac{g}{2}\right){\bar{F}}_{j}^{\prime \prime }{\tilde{u}}_{j}^{\left(2\right)}\\ & & +\left(\frac{1}{6}{\left[{\tilde{u}}_{j}^{\left(1\right)}\right]}^{3}+\frac{g}{4}{\left[{\tilde{u}}_{j}^{\left(1\right)}\right]}^{2}+\frac{g^{2}}{6}{\tilde{u}}_{j}^{\left(1\right)}+\frac{g^{3}}{24}\right){\bar{F}}_{j}^{\prime \prime \prime }]T^{\left(0\right)}\\ & & +[\left(\;{\bar{F}}_{j}^{\prime 2}+{\bar{F}}_{j}{\bar{F}}_{j}^{\prime \prime }\right)\frac{{\tilde{u}}_{j}^{\left(2\right)}}{2}+\left(3{\bar{F}}_{j}^{\prime }{\bar{F}}_{j}^{\prime \prime }+{\bar{F}}_{j}{\bar{F}}_{j}^{\prime \prime \prime }\right)\frac{{\left[{\tilde{u}}_{j}^{\left(1\right)}\right]}^{2}}{4}\\ & & +g\left(2{\bar{F}}_{j}^{\prime }{\bar{F}}_{j}^{\prime \prime }+{\bar{F}}_{j}{\bar{F}}_{j}^{\prime \prime \prime }\right)\frac{{\tilde{u}}_{j}^{\left(1\right)}}{3}+\frac{g^{2}}{6}{\bar{F}}_{j}^{\prime }{\bar{F}}_{j}^{\prime \prime }+\frac{g^{2}}{8}{\bar{F}}_{j}{\bar{F}}_{j}^{\prime \prime \prime }]{T^{\left(0\right)}}^{2}\\ & & +\left[\left(g+4{\tilde{u}}_{j}^{\left(1\right)}\right){\bar{F}}_{j}^{\prime 3}+\left(8g+16{\tilde{u}}_{j}^{\left(1\right)}\right){\bar{F}}_{j}{\bar{F}}_{j}^{\prime }{\bar{F}}_{j}^{\prime \prime }+\left(3g+4{\tilde{u}}_{j}^{\left(1\right)}\right){\bar{F}}_{j}^{2}{\bar{F}}_{j}^{\prime \prime \prime }\right]\frac{{T^{\left(0\right)}}^{3}}{24}\\ & & +\left[{\bar{F}}_{j}{\bar{F}}_{j}^{\prime 3}+4{\bar{F}}_{j}^{2}{\bar{F}}_{j}^{\prime }{\bar{F}}_{j}^{\prime \prime }+{\bar{F}}_{j}^{3}{\bar{F}}_{j}^{\prime \prime \prime }\right]\frac{{T^{\left(0\right)}}^{4}}{24}\\ & & +\left[{\bar{F}}_{j}^{\prime }{\tilde{u}}_{j}^{\left(2\right)}+\frac{{\bar{F}}_{j}^{\prime \prime }}{2}{\left[{\tilde{u}}_{j}^{\left(1\right)}\right]}^{2}+g\frac{{\bar{F}}_{j}^{\prime \prime }}{2}{\tilde{u}}_{j}^{\left(1\right)}+\frac{g^{2}}{6}{\bar{F}}_{j}^{\prime \prime }\right]T^{\left(1\right)}\\ & & +\left[\frac{2g}{3}{\bar{F}}_{j}^{\prime \prime }{\bar{F}}_{j}+{\bar{F}}_{j}^{\prime 2}{\tilde{u}}_{j}^{\left(1\right)}+{\bar{F}}_{j}^{\prime \prime }{\bar{F}}_{j}{\tilde{u}}_{j}^{\left(1\right)}+\frac{g}{3}{\bar{F}}_{j}^{\prime 2}\right]T^{\left(0\right)}T^{\left(1\right)}\\ & & +\frac{{\bar{F}}_{j}}{2}\left(\;{\bar{F}}_{j}^{\prime \prime }{\bar{F}}_{j}+{\bar{F}}_{j}^{\prime 2}\right){T^{\left(0\right)}}^{2}T^{\left(1\right)}\\ & & +\left\{\left({\tilde{u}}_{j}^{\left(1\right)}+\frac{g}{2}\right)T^{\left(2\right)}+\frac{{\bar{F}}_{j}}{2}\left[{T^{\left(1\right)}}^{2}+2T^{\left(0\right)}T^{\left(2\right)}\right]\right\}{\bar{F}}_{j}^{\prime }+{\bar{F}}_{j}T^{\left(3\right)}. \end{array}$$

## Appendix B: Fixed-point expansion (LIF model)

In the case of the LIF neuron (see Eq. (2)), the fixed point of the event-driven map reads

(67) $$u_{j-1}=e^{-\tau }u_{j}+\chi ,$$

where

(68) $$\chi =a\left(1-e^{-\tau }\right)+g\frac{e^{-\tau }-e^{-\alpha \tau }}{\alpha -1}\left(E+\frac{P}{\alpha -1}\right)-g\frac{\tau }{\alpha -1}e^{-\alpha \tau }P.$$

Its solution is

(69) $$u_{j}=\chi \frac{1-e^{-NT+j\tau }}{1-e^{-\tau }}.$$

By expanding Eq. (69) for j=0 and for a generic *j*, one can derive perturbative expressions for the period *T* and the membrane potential, respectively. Let us start by substituting the expressions (17), (60), (61), (62) in Eq. (68). This leads to the expansion

(70) $$\chi =\left(a+\frac{g}{T}\right)\left[\tau -\frac{\tau^{2}}{2}+\frac{\tau^{3}}{6}-\frac{\tau^{4}}{24}\right]+a\frac{\tau^{5}}{120}+\frac{g}{T}\frac{\tau^{5}}{720}K(\alpha )+O\left(1/N^{4}\right),$$

where

(71) $$K(\alpha )=\frac{360\alpha^{6}-722\alpha^{5}+363\alpha^{4}+5\alpha^{2}-12\alpha +6}{{\left(\alpha -1\right)}^{2}},$$

accounts for the dependence on the field dynamics. Now, with the help of Eqs. (17), (70) and expanding the exponential terms up to the fourth order, we obtain a closed equation for the interspike interval,

(72) $$\begin{array}{rcl} u_{0} & = & 1=\left(a+\frac{g}{T^{\left(0\right)}}\right)\left(1-e^{-T^{\left(0\right)}}\right)+\frac{T^{\left(1\right)}}{N}\xi \left(T^{\left(0\right)}\right)\\ & & +\frac{1}{N^{2}}\left[T^{\left(2\right)}\xi \left(T^{\left(0\right)}\right)+T^{\left(1\right)}W_{1}^{\left(2\right)}\right]\\ & & +\frac{1}{N^{3}}\left[T^{\left(3\right)}\xi \left(T^{\left(0\right)}\right)+T^{\left(1\right)}W_{1}^{\left(3\right)}+T^{\left(2\right)}W_{2}^{\left(3\right)}\right]\\ & & +\frac{1}{N^{4}}\left[T^{\left(4\right)}\xi \left(T^{\left(0\right)}\right)+\zeta \left(T^{\left(0\right)}\right)+T^{\left(1\right)}W_{1}^{\left(4\right)}+T^{\left(2\right)}W_{2}^{\left(4\right)}+T^{\left(3\right)}W_{3}^{\left(4\right)}\right],\; \end{array}$$

where

$$\begin{array}{rcl} \zeta \left(T^{\left(0\right)}\right) & = & -\left(1-e^{-T^{\left(0\right)}}\right)\frac{g}{120}{T^{\left(0\right)}}^{3}\left(1-\frac{K(\alpha )}{6}\right),\\ \xi \left(T^{\left(0\right)}\right) & = & \left(a+\frac{g}{T^{\left(0\right)}}\right)e^{-T^{\left(0\right)}}-\frac{g}{{T^{\left(0\right)}}^{2}}\left(1-e^{-T^{\left(0\right)}}\right), \end{array}$$

while $W_{k}^{\left(h\right)}$ identifies a term of order $1/N^{h}$ that is multiplied by $T^{\left(k\right)}$. Since, while proceeding from lower- to higher-order terms, we find that $T^{\left(k\right)}=0$ (for k<4), it is not necessary to give the explicit expression of the $W_{k}^{\left(h\right)}$ functions as they do not contribute at all.

One can equivalently expand $u_{j}$

(73) $$\begin{array}{rcl} u_{j} & = & u_{j}^{\left(0\right)}+\frac{u_{j}^{\left(1\right)}}{N}+\frac{u_{j}^{\left(2\right)}}{N^{2}}+\frac{u_{j}^{\left(3\right)}}{N^{3}}+\frac{u_{j}^{\left(4\right)}}{N^{4}}\\ & = & \left(a+\frac{g}{T^{\left(0\right)}}\right)\left(1-e^{T^{\left(0\right)}(\frac{j}{N}-1)}\right)+\frac{T^{\left(1\right)}}{N}\varsigma \left(T^{\left(0\right)}\right)\\ & & +\frac{1}{N^{2}}\left[T^{\left(2\right)}\varsigma \left(T^{\left(0\right)}\right)+T^{\left(1\right)}Z_{1}^{\left(2\right)}\right]+\frac{1}{N^{3}}\left[T^{\left(3\right)}\varsigma \left(T^{\left(0\right)}\right)+T^{\left(1\right)}Z_{1}^{\left(3\right)}+T^{\left(2\right)}Z_{2}^{\left(3\right)}\right]\\ & & +\frac{1}{N^{4}}\left[\varsigma \left(T^{\left(0\right)}\right)T^{\left(4\right)}+\sigma \left(T^{\left(0\right)}\right)+T^{\left(1\right)}Z_{1}^{\left(4\right)}+T^{\left(2\right)}Z_{2}^{\left(4\right)}+T^{\left(3\right)}Z_{3}^{\left(4\right)}\right], \end{array}$$

where

$$\begin{array}{rcl} \varsigma \left(T^{\left(0\right)}\right) & = & -\left(a+\frac{g}{T^{\left(0\right)}}\right)e^{T^{\left(0\right)}(\frac{j}{N}-1)}\left(\frac{j}{N}-1\right)-\frac{g}{{T^{\left(0\right)}}^{2}}\left[1-e^{T^{\left(0\right)}(\frac{j}{N}-1)}\right],\\ \sigma \left(T^{\left(0\right)}\right) & = & \frac{g}{120}\left(1-e^{T^{\left(0\right)}(\frac{j}{N}-1)}\right){T^{\left(0\right)}}^{3}\left(\frac{K}{6}-1\right), \end{array}$$

while we do not provide explicit expressions for $Z_{k}^{\left(h\right)}$ as they turn out to be irrelevant.

Now we are in the position to analyse the different orders.

### B.1 Zeroth order

By assembling the terms of order one in Eq. (72), we obtain

(74) $$\left(a+\frac{g}{T^{\left(0\right)}}\right)\left(1-e^{-T^{\left(0\right)}}\right)=1.$$

This is an implicit definition of the asymptotic interspike time $T^{\left(0\right)}$

(75) $$T^{\left(0\right)}=\ln\left(\frac{aT^{\left(0\right)}+g}{T^{\left(0\right)}(a-1)+g}\right).$$

Analogously, we can find an explicit equation for the membrane potential by assembling the terms of order one in Eq. (73)

(76) $$u_{j}^{\left(0\right)}=\left(a+\frac{g}{T^{\left(0\right)}}\right)\left[1-e^{T^{\left(0\right)}(\frac{j}{N}-1)}\right].$$

In the thermodynamic limit, the solution for $u_{j}^{\left(0\right)}$ becomes

(77) $$U^{\left(0\right)}(x)=\left(a+\frac{g}{T^{\left(0\right)}}\right)\left[1-e^{T^{\left(0\right)}(x-1)}\right],$$

which coincides with Eq. (23) with $F=a-U^{\left(0\right)}$.

### B.2 From first to third order

By separately assembling the terms of order $1/N^{h}$ (for h=1,2,3) in Eq. (72), we obtain

(78) $$T^{\left(h\right)}\xi \left(T^{\left(0\right)}\right)=0,$$

which implies that $T^{\left(h\right)}=0$ since ξ≠0. Moreover, by assembling the terms of order $1/N^{h}$ in Eq. (73), we obtain

(79) $$u_{j}^{\left(h\right)}=\varsigma \left(T^{\left(0\right)}\right)T^{\left(h\right)},\;j=1,\ldots ,N,$$

which thereby implies that first-, second- and third-order corrections vanish also for the membrane potential.

### B.3 Fourth order

The order which reveals a different scenario is the fourth one. By assembling the terms of order $1/N^{4}$ in Eq. (72), we obtain

(80) $$T^{\left(4\right)}=-\frac{\zeta (T^{\left(0\right)})}{\xi (T^{\left(0\right)})},$$

whose explicit expression is reported in Eq. (5). By analogously assembling the terms of order $1/N^{4}$ in Eq. (73), we obtain

(81) $$u_{j}^{\left(4\right)}=\varsigma \left(T^{\left(0\right)}\right)T^{\left(4\right)}+\sigma \left(T^{\left(0\right)}\right),$$

which becomes, in the thermodynamic limit,

$$\begin{array}{rcl} U^{\left(4\right)}(x) & = & -\left(a+\frac{g}{T^{\left(0\right)}}\right)T^{\left(4\right)}e^{T^{\left(0\right)}(x-1)}(x-1)-g\frac{T^{\left(4\right)}}{{T^{\left(0\right)}}^{2}}\left[1-e^{T^{\left(0\right)}(x-1)}\right]\\ & & +\frac{g}{120}\left(1-e^{T^{\left(0\right)}(x-1)}\right){T^{\left(0\right)}}^{3}\left(\frac{K(\alpha )}{6}-1\right). \end{array}$$

## Appendix C: Expansion in tangent space around the fixed point

### C.1 Introduction

The auxiliary variables required to complete the definition of the tangent-space evolution rule (35), (36), (37), (38) are as follows:

$$\begin{array}{rcl} Z_{1} & = & \left(1-\alpha \tau +\frac{\alpha^{2}}{2}\tau^{2}-\frac{\alpha^{3}}{6}\tau^{3}+\frac{\alpha^{4}}{24}\tau^{4}\right),\\ Z_{2} & = & \left(-\alpha +\alpha^{2}\tau -\frac{\alpha^{3}}{2}\tau^{2}+\frac{\alpha^{4}}{6}\tau^{3}\right),\;Z_{3}=\left(\tau -\alpha \tau^{2}+\frac{\alpha^{2}}{2}\tau^{3}-\frac{\alpha^{3}}{6}\tau^{4}\right),\\ Z_{4} & = & \left(1-2\alpha \tau +\frac{3}{2}\alpha^{2}\tau^{2}-\frac{2}{3}\alpha^{3}\tau^{3}+\frac{5}{24}\alpha^{4}\tau^{4}\right),\\ W_{1} & = & \{1+{\bar{F}}_{j}^{\prime }\tau +\frac{\tau^{2}}{2}\left(\;{\bar{F}}_{j}^{\prime \prime }{\bar{F}}_{j}+{\bar{F}}_{j}^{\prime 2}\right)\\ & & +\frac{\tau^{3}}{6}\left[\;{\bar{F}}_{j}^{\prime \prime \prime }{\bar{F}}_{j}^{2}+{\bar{F}}_{j}^{\prime \prime }\left(4{\bar{F}}_{j}^{\prime }{\bar{F}}_{j}+g\dot{\tilde{E}}\right)+{\bar{F}}_{j}^{2}{\bar{F}}_{j}^{\prime }\right]\},\\ W_{2} & = & \left[g\tau +\frac{\tau^{2}}{2}g{\bar{F}}_{j}^{\prime }+\frac{\tau^{3}}{6}\left(2g{\bar{F}}_{j}^{\prime \prime }{\bar{F}}_{j}+g{\bar{F}}_{j}^{2}\right)\right],\\ W_{3} & = & \left[g\frac{\tau^{2}}{2}+\frac{\tau^{3}}{6}\left(g{\bar{F}}_{j}^{\prime }-g\alpha \right)\right],\\ W_{4} & = & \{{\bar{F}}_{j}+\left[\;{\bar{F}}_{j}^{\prime }{\bar{F}}_{j}+g\dot{\tilde{E}}\right]\tau +\left[\;{\bar{F}}_{j}^{\prime \prime }{\bar{F}}_{j}^{2}+{\left(\;{\bar{F}}_{j}^{\prime }\right)}^{2}{\bar{F}}_{j}+g{\bar{F}}_{j}^{\prime }\dot{\tilde{E}}-g\alpha (\dot{\tilde{E}}+\tilde{P})\right]\frac{\tau^{2}}{2}\\ & & +\left[{\left(\;{\bar{F}}_{j}^{\prime }\right)}^{3}{\bar{F}}_{j}+4{\bar{F}}_{j}^{\prime }{\bar{F}}_{j}^{\prime \prime }{\bar{F}}_{j}^{2}+{\bar{F}}_{j}^{\prime \prime \prime }{\bar{F}}_{j}^{3}+g\alpha \left(2\alpha -{\bar{F}}_{j}^{\prime }\right)\tilde{P}\right]\frac{\tau^{3}}{6}\},\\ W_{5} & = & \{g\tau -g\left[\frac{1}{2}\left(\;{\bar{F}}_{1}^{\prime }+\alpha \right)+\frac{1}{{\bar{F}}_{1}}g\dot{\tilde{E}}\right]\tau^{2}+\frac{g}{{\bar{F}}_{1}}[g\alpha \left(\dot{\tilde{E}}+\frac{\tilde{P}}{2}\right)\\ & & +\frac{{\bar{F}}_{1}}{6}\left(\alpha^{2}-5{\bar{F}}_{1}^{\prime 2}-{\bar{F}}_{1}^{\prime \prime }{\bar{F}}_{1}+2\alpha {\bar{F}}_{1}^{\prime }\right)+\frac{1}{{\bar{F}}_{1}}{\left(\;{\bar{F}}_{1}^{\prime }{\bar{F}}_{1}+g\dot{\tilde{E}}\right)}^{2}]\tau^{3}\},\\ W_{6} & = & \left\{\frac{g}{2}\tau^{2}-\frac{g}{2}\tau^{3}\left[\frac{2}{3}\left(\alpha +{\bar{F}}_{1}^{\prime }\right)+\frac{1}{{\bar{F}}_{1}}g\dot{\tilde{E}}\right]\right\},\\ W_{7} & = & \{1-\frac{g}{{\bar{F}}_{1}}\tau \dot{\tilde{E}}+\tau^{2}\left[\frac{g\alpha }{2{\bar{F}}_{1}}(\dot{\tilde{E}}+\tilde{P})+\frac{1}{{\bar{F}}_{1}^{2}}{\left(\;{\bar{F}}_{1}^{\prime }{\bar{F}}_{1}+g\dot{\tilde{E}}\right)}^{2}-{\bar{F}}_{1}^{\prime 2}-\frac{3}{2}\frac{g}{{\bar{F}}_{1}}{\bar{F}}_{1}^{\prime }\dot{\tilde{E}}\right]\\ & & -\tau^{3}[\frac{1}{3{\bar{F}}_{1}}g\alpha \left(\alpha +{\bar{F}}_{1}^{\prime }\right)\tilde{P}+\frac{1}{{\bar{F}}_{1}^{2}}g\alpha \dot{\tilde{E}}\left(\frac{1}{2}{\bar{F}}_{1}^{\prime }{\bar{F}}_{1}+g\tilde{P}\right)\\ & & +\frac{1}{{\bar{F}}_{1}^{3}}g^{2}{\dot{\tilde{E}}}^{3}+\frac{1}{{\bar{F}}_{1}^{2}}g^{2}{\dot{\tilde{E}}}^{2}\left(\alpha +{\bar{F}}_{1}^{\prime }\right)-\frac{2}{3}g{\bar{F}}_{1}^{\prime \prime }\dot{\tilde{E}}]\}, \end{array}$$

where the dependence of *τ* on *n* has been dropped, since we are considering a linearisation around the splay state.

In order to find the Floquet eigenvalue $\mu_{k}$, one should substitute the Ansätze (39) into Eqs. (35), (36). This allows to find explicit expressions for *δP* and *δE* as a function of $\mu_{k}$, *τ* and *δτ*, namely

(82) $$\delta P=-\frac{\alpha^{2}}{\mu_{k}-1}\left[1-\frac{\alpha \tau }{2}\frac{\mu_{k}+1}{\mu_{k}-1}+\alpha^{2}\tau^{2}M_{k}-\frac{\alpha^{3}\tau^{3}}{2}\frac{\mu_{k}(\mu_{k}+1)}{{\left(\mu_{k}-1\right)}^{3}}\right]\frac{\delta \tau }{T},$$

(83) $$\delta E=-\frac{\alpha }{\mu_{k}-1}\left[\frac{\alpha \tau }{2}\frac{\left(\mu_{k}+1\right)}{\mu_{k}-1}-2\alpha^{2}\tau^{2}M_{k}-\frac{3\alpha^{3}\tau^{3}}{2}\frac{\mu_{k}(\mu_{k}+1)}{{\left(\mu_{k}-1\right)}^{3}}\right]\frac{\delta \tau }{T},$$

where we have introduced the shorthand notation

(84) $$M_{k}=\frac{\mu_{k}^{2}+10\mu_{k}+1}{12{\left(\mu_{k}-1\right)}^{2}}.$$

By substituting *δP* and *δE*, as given by (82) and (83), into Eq. (38), we can express *δτ* directly in terms of $\delta u_{1}$

(85) $$\delta \tau =-\left\{{\bar{F}}_{1}+\frac{g\alpha^{2}}{T}M_{k}\tau^{2}-\frac{g\alpha^{2}}{T}\left[\frac{{\bar{F}}_{1}^{\prime }}{12}\frac{\left(\mu_{k}+5\right)}{{\left(\mu_{k}-1\right)}^{2}}+\alpha \frac{\left(\mu_{k}+1\right)}{{\left(\mu_{k}-1\right)}^{3}}\right]\mu_{k}\tau^{3}\right\}\frac{\delta u_{1}}{{\bar{F}}_{1}^{2}},$$

where we exploited the equality ${\bar{F}}_{j}\equiv {\bar{F}}_{j}+\frac{g}{T}$, which follows from the fact that in the thermodynamic limit $\tilde{E}=\frac{1}{T}$.

By inserting the expressions in Eqs. (82), (83), (85) into Eq. (37), we find a single equation for the eigenvalues and eigenvectors,

$$\begin{array}{rcl} \mu_{k}\delta u_{j-1} & = & \left\{1+{\bar{F}}_{j}^{\prime }\tau +\left[\;{\bar{F}}_{j}^{\prime \prime }{\bar{F}}_{j}+{\bar{F}}_{j}^{\prime 2}\right]\frac{\tau^{2}}{2}+\left[\;{\bar{F}}_{j}^{\prime \prime \prime }{\bar{F}}_{j}^{2}+4{\bar{F}}_{j}^{\prime }{\bar{F}}_{j}^{\prime \prime }{\bar{F}}_{j}+{\bar{F}}_{j}^{\prime 3}\right]\frac{\tau^{3}}{6}\right\}\delta u_{j}\\ & & -\{{\bar{F}}_{j}+{\bar{F}}_{j}^{\prime }{\bar{F}}_{j}\tau +\left[\frac{{\bar{F}}_{j}^{\prime \prime }}{2}{\bar{F}}_{j}^{2}+\frac{{\bar{F}}_{j}^{\prime 2}}{2}{\bar{F}}_{j}+\frac{g\alpha^{2}}{T}M_{k}\left(\frac{{\bar{F}}_{j}}{{\bar{F}}_{1}}-1\right)\right]\tau^{2}\\ & & +[\frac{{\bar{F}}_{j}^{\prime 3}}{6}{\bar{F}}_{j}+\frac{2}{3}{\bar{F}}_{j}^{\prime }{\bar{F}}_{j}^{\prime \prime }{\bar{F}}_{j}^{2}+\frac{{\bar{F}}_{j}^{\prime \prime \prime }}{6}{\bar{F}}_{j}^{3}\\ & & +\frac{g\alpha^{2}}{T}\frac{5\mu_{k}+1}{12{\left(\mu_{k}-1\right)}^{2}}\left(\frac{{\bar{F}}_{j}}{{\bar{F}}_{1}}{\bar{F}}_{1}^{\prime }-{\bar{F}}_{j}^{\prime }\right)+\frac{g\alpha^{3}}{T}\frac{\mu_{k}(\mu_{k}+1)}{{\left(\mu_{k}-1\right)}^{3}}\left(1-\frac{{\bar{F}}_{j}}{{\bar{F}}_{1}}\right)\\ & & +\frac{g\alpha^{2}}{T}\frac{{\bar{F}}_{j}}{{\bar{F}}_{1}}\left(\;{\bar{F}}_{j}^{\prime }-{\bar{F}}_{1}^{\prime }\right)M_{k}]\tau^{3}\}\frac{\delta u_{1}}{{\bar{F}}_{1}}. \end{array}$$

By now substituting the $\mu_{k}$ expansion (40) and retaining the leading terms, we obtain Eq. (41).

### C.2 N→∞ limit

Once the continuous variables (44) have been introduced, it is necessary to estimate U(1/N) and δU(1/N) by expanding such variables around zero. By inserting the resulting expansion for U(1/N) into the expressions for ${\bar{F}}_{1}$ and ${\bar{F}}_{1}$, we obtain, respectively,

$$\begin{array}{rcl} F\left(U\left(\frac{1}{N}\right)\right) & = & F\left(U(0)\right)+{\frac{F^{\prime }(U(0))}{N}\frac{dU}{dx}|}_{0}\\ & & +{\frac{F^{\prime }(U(0))}{2N^{2}}\frac{d^{2}U}{dx^{2}}|}_{0}+\frac{F^{\prime \prime }(U(0))}{2}{\left({\frac{1}{N}\frac{dU}{dx}|}_{0}\right)}^{2}\\ & & +{\frac{F^{\prime \prime }(U(0))}{2N^{3}}\frac{dU}{dx}|}_{0}{\frac{d^{2}U}{dx^{2}}|}_{0}+\frac{F^{\prime \prime \prime }(U(0))}{6}{\left({\frac{1}{N}\frac{dU}{dx}|}_{0}\right)}^{3}\\ & & +\frac{F^{\prime }(U(0))}{6N^{3}}\frac{d^{3}U}{dx^{3}}{|}_{0}+O\left(\frac{1}{N^{4}}\right),\\ \frac{1}{F(U(\frac{1}{N}))} & \equiv & \frac{1}{F(U(\frac{1}{N}))+\frac{g}{T}}=\frac{1}{F(U(0))}+\frac{1}{N}\frac{TF^{\prime }(U(0))}{F(U(0))}\\ & & +\frac{1}{2N^{2}}\frac{{\left[TF^{\prime }(U(0))\right]}^{2}}{F(U(0))}-\frac{1}{2N^{2}}F^{\prime \prime }\left(U(0)\right)T^{2}\\ & & +\frac{T^{3}}{6N^{3}}[F^{\prime \prime \prime }\left(U(0)\right)F\left(U(0)\right)-2F^{\prime }\left(U(0)\right)F^{\prime \prime }\left(U(0)\right)\\ & & -\frac{g}{T^{2}}{\left(\frac{F^{\prime }(U(0))}{F(U(0))}\right)}^{2}-5\frac{{\left[F^{\prime }(U(0))\right]}^{3}}{F(U(0))}+6\frac{F^{\prime }(U(0))}{F(U(0))}]+O\left(\frac{1}{N^{4}}\right). \end{array}$$

An analogous procedure for δU(1/N) leads to

$$\begin{array}{rcl} \delta U(1/N) & = & e^{i\phi_{k}}\left[\Theta^{\left(0\right)}\left(\frac{1}{N}\right)+\frac{\Theta^{\left(1\right)}(\frac{1}{N})}{N}+\frac{\Theta^{\left(2\right)}(\frac{1}{N})}{N^{2}}\right]\\ & & +\Pi^{\left(0\right)}\left(\frac{1}{N}\right)+\frac{\Pi^{\left(1\right)}(\frac{1}{N})}{N}+\frac{\Pi^{\left(2\right)}(\frac{1}{N})}{N^{2}}+O\left(\frac{1}{N^{3}}\right)\\ & \equiv & C^{\left(0\right)}+\frac{C^{\left(1\right)}}{N}+\frac{C^{\left(2\right)}}{N^{2}}+O\left(\frac{1}{N^{3}}\right), \end{array}$$

where $C^{\left(0\right)}$, $C^{\left(1\right)}$, $C^{\left(2\right)}$ are defined according to the following equations:

$$\begin{array}{rcl} C^{\left(0\right)} & = & e^{i\phi_{k}}\Theta^{\left(0\right)}(0)+\Pi^{\left(0\right)}(0),\\ C^{\left(1\right)} & = & e^{i\phi_{k}}\left[{\frac{d\Theta^{\left(0\right)}}{dx}|}_{0}+\Theta^{\left(1\right)}(0)\right]+{\frac{d\Pi^{\left(0\right)}}{dx}|}_{0}+\Pi^{\left(1\right)}(0),\\ C^{\left(2\right)} & = & e^{i\phi_{k}}\left[{\frac{1}{2}\frac{d^{2}\Theta^{\left(0\right)}}{dx^{2}}|}_{0}+{\frac{d\Theta^{\left(1\right)}}{dx}|}_{0}+\Theta^{\left(2\right)}(0)\right]+\frac{1}{2}{\frac{d^{2}\Pi^{\left(0\right)}}{dx^{2}}|}_{0}+{\frac{d\Pi^{\left(1\right)}}{dx}|}_{0}+\Pi^{\left(2\right)}(0). \end{array}$$

We now expand δU(1/N) up to the order $O(\frac{1}{N^{3}})$, thus neglecting higher orders because they contribute to the definition of Π variable and we need terms at least of order $O(\frac{1}{N^{3}})$ (one order lower than needed to define Θ). By inserting the Ansatz (42) and the previous expansions in Eq. (41), we finally obtain a closed equation for the eigenvalues and eigenvectors,

(86) $$\begin{array}{rcl} & & e^{i\phi_{k}}\{\Pi^{\left(0\right)}+\left[\Pi^{\left(1\right)}-{\Pi^{\left(0\right)}}^{\prime }+\Pi^{\left(0\right)}\Gamma^{\left(1\right)}\right]\frac{1}{N}\\ & & \;+\left[\frac{{\Pi^{\left(0\right)}}^{\prime \prime }}{2}-{\Pi^{\left(1\right)}}^{\prime }+\Pi^{\left(2\right)}-{\Pi^{\left(0\right)}}^{\prime }\Gamma^{\left(1\right)}+\Pi^{\left(1\right)}\Gamma^{\left(1\right)}+\Pi^{\left(0\right)}\Gamma^{\left(2\right)}\right]\frac{1}{N^{2}}\\ & & \;+[\frac{{\Pi^{\left(1\right)}}^{\prime \prime }}{2}-\frac{{\Pi^{\left(0\right)}}^{\prime \prime \prime }}{6}-{\Pi^{\left(2\right)}}^{\prime }+\Pi^{\left(3\right)}+\frac{{\Pi^{\left(0\right)}}^{\prime \prime }\Gamma^{\left(1\right)}}{2}-{\Pi^{\left(1\right)}}^{\prime }\Gamma^{\left(1\right)}+\Pi^{\left(2\right)}\Gamma^{\left(1\right)}\\ & & \;-{\Pi^{\left(0\right)}}^{\prime }\Gamma^{\left(2\right)}+\Pi^{\left(1\right)}\Gamma^{\left(2\right)}+\Pi^{\left(0\right)}\Gamma^{\left(3\right)}]\frac{1}{N^{3}}\}\\ & & \;-\Pi^{\left(0\right)}-\left[\Pi^{\left(1\right)}+\Pi^{\left(0\right)}A^{\left(1\right)}\right]\frac{1}{N}-\left[\Pi^{\left(2\right)}+\Pi^{\left(0\right)}A^{\left(2\right)}+\Pi^{\left(1\right)}A^{\left(1\right)}\right]\frac{1}{N^{2}}\\ & & \;-\left[\Pi^{\left(3\right)}+\Pi^{\left(0\right)}A^{\left(3\right)}+\Pi^{\left(1\right)}A^{\left(2\right)}+\Pi^{\left(2\right)}A^{\left(1\right)}\right]\frac{1}{N^{3}}\\ & & \;+C^{\left(0\right)}B^{\left(0\right)}+\left[C^{\left(0\right)}B^{\left(1\right)}+C^{\left(1\right)}B^{\left(0\right)}\right]\frac{1}{N}\\ & & \;+\left[C^{\left(0\right)}B^{\left(2\right)}+C^{\left(1\right)}B^{\left(1\right)}+C^{\left(2\right)}B^{\left(0\right)}\right]\frac{1}{N^{2}}+\frac{B}{N^{3}}\\ & & \;=e^{i\phi_{k}j}\{\left[{\Theta^{\left(0\right)}}^{\prime }-\Theta^{\left(0\right)}\Gamma^{\left(1\right)}+\Theta^{\left(0\right)}A^{\left(1\right)}\right]\frac{1}{N}\\ & & \;+[\left(\Theta^{\left(0\right)}A^{\left(2\right)}-\frac{{\Theta^{\left(0\right)}}^{\prime \prime }}{2}\right)+{\Theta^{\left(1\right)}}^{\prime }+{\Theta^{\left(0\right)}}^{\prime }\Gamma^{\left(1\right)}-\Theta^{\left(1\right)}\Gamma^{\left(1\right)}-\Theta^{\left(0\right)}\Gamma^{\left(2\right)}\\ & & \;+\Theta^{\left(1\right)}A^{\left(1\right)}]\frac{1}{N^{2}}+[\left(\frac{-{\Theta^{\left(1\right)}}^{\prime \prime }}{2}+\Theta^{\left(1\right)}A^{\left(2\right)}\right)+\left(\frac{{\Theta^{\left(0\right)}}^{\prime \prime \prime }}{6}+\Theta^{\left(0\right)}A^{\left(3\right)}\right)\\ & & \;+{\Theta^{\left(2\right)}}^{\prime }-\frac{{\Theta^{\left(0\right)}}^{\prime \prime }\Gamma^{\left(1\right)}}{2}+{\Theta^{\left(1\right)}}^{\prime }\Gamma^{\left(1\right)}-\Theta^{\left(2\right)}\Gamma^{\left(1\right)}+{\Theta^{\left(0\right)}}^{\prime }\Gamma^{\left(2\right)}\\ & & \;-\Theta^{\left(1\right)}\Gamma^{\left(2\right)}-\Theta^{\left(0\right)}\Gamma^{\left(3\right)}+\Theta^{\left(2\right)}A^{\left(1\right)}]\frac{1}{N^{3}}\}, \end{array}$$

where we have introduced the shorthand notation ℬ in order to characterise a term of order $O(\frac{1}{N^{3}})$, whose explicit expression is not necessary, since it turns out to contribute to the definition of the Π variable, and it is, therefore, one order beyond what we need. Moreover, notice that the terms appearing within round brackets in the rhs of the above equation can be shown to be zero due to exact algebraic cancellations that emerge from the solution of the equation order by order. Finally,

$$\begin{array}{rcl} A^{\left(1\right)}\left(U(x)\right) & = & TF^{\prime }\left(U(x)\right),\\ A^{\left(2\right)}\left(U(x)\right) & = & \frac{T^{2}}{2}\left\{F^{\prime \prime }\left(U(x)\right)F\left(U(x)\right)+{\left[F^{\prime }\left(U(x)\right)\right]}^{2}\right\},\\ A^{\left(3\right)}\left(U(x)\right) & = & \frac{T^{3}}{6}\{F^{\prime \prime \prime }\left(U(x)\right){\left[F\left(U(x)\right)\right]}^{2}\\ & & +4F^{\prime }\left(U(x)\right)F^{\prime \prime }\left(U(x)\right)F\left(U(x)\right)+{\left[F^{\prime }\left(U(x)\right)\right]}^{3}\},\\ B^{\left(0\right)}\left(U(x)\right) & = & \frac{F(U(x))}{F(U(0))},\\ B^{\left(1\right)}\left(U(x)\right) & = & \left[TF^{\prime }\left(U(x)\right)+TF^{\prime }\left(U(0)\right)\right]\frac{F(U(x))}{F(U(0))},\\ B^{\left(2\right)}\left(U(x)\right) & = & T^{2}\{-\frac{g}{T}\alpha^{2}\frac{e^{2i\phi_{k}}+10e^{i\phi_{k}}+1}{12{\left(e^{i\phi_{k}}-1\right)}^{2}}\frac{F(U(0))-F(U(x))}{{\left[F(U(0))\right]}^{2}}\\ & & +\frac{F^{\prime \prime }(U(x))}{2}\frac{{\left[F(U(x))\right]}^{2}}{F(U(0))}+\frac{{\left[F^{\prime }(U(x))\right]}^{2}}{2}\frac{F(U(x))}{F(U(0))}\}\\ & & +\left\{TF^{\prime }\left(U(x)\right)TF^{\prime }\left(U(0)\right)+\frac{{\left[TF^{\prime }(U(0))\right]}^{2}}{2}\right\}\frac{F(U(x))}{F(U(0))}\\ & & -\frac{T^{2}}{2}F^{\prime \prime }\left(U(0)\right)F\left(U(x)\right). \end{array}$$

## Competing interests

The authors declare that they have no competing interests.

## Authors’ contributions

SO carried out the analytical calculations and drafted the manuscript. AT participated in the design of the study and in the drawing up of the article. AP conceived the study and participated in its design and coordination. All authors read and approved the final manuscript.
